# Development of Medical Countermeasures to Middle East Respiratory Syndrome Coronavirus

**DOI:** 10.3201/eid2207.160022

**Published:** 2016-07

**Authors:** Timothy M. Uyeki, Karl J. Erlandson, George Korch, Michael O’Hara, Michael Wathen, Jean Hu-Primmer, Sally Hojvat, Erik J. Stemmy, Armen Donabedian

**Affiliations:** Centers for Disease Control and Prevention, Atlanta, Georgia, USA (T.M. Uyeki);; Office of the Assistant Secretary of Preparedness and Response, Washington, DC, USA (K.J. Erlandson, G. Korch, M. O’Hara, M. Wathen, J. Hu-Primmer, A. Donabedian);; Food and Drug Administration, Silver Spring, Maryland, USA (S. Hojvat);; National Institutes of Health, Rockville, Maryland, USA (E.J. Stemmy)

**Keywords:** Middle East respiratory syndrome coronavirus, MERS-CoV, medical countermeasures, animal models, non-human primates, nonhuman primates, diagnostics, therapeutics, antivirals, monoclonal, polyclonal, antibodies, vaccines, viruses, respiratory infections

## Abstract

Preclinical development of and research on potential Middle East respiratory syndrome coronavirus (MERS-CoV) medical countermeasures remain preliminary; advancements are needed before most countermeasures are ready to be tested in human clinical trials. Research priorities include standardization of animal models and virus stocks for studying disease pathogenesis and efficacy of medical countermeasures; development of MERS-CoV diagnostics; improved access to nonhuman primates to support preclinical research; studies to better understand and control MERS-CoV disease, including vaccination studies in camels; and development of a standardized clinical trial protocol. Partnering with clinical trial networks in affected countries to evaluate safety and efficacy of investigational therapeutics will strengthen efforts to identify successful medical countermeasures.

From September 2012 through April 27, 2016, a total of 1,728 laboratory-confirmed Middle East respiratory syndrome coronavirus (MERS-CoV) infections, leading to 624 deaths (36% case-fatality proportion), had been reported to the World Health Organization (WHO) (*1*). Most infections (75%) have been identified in Saudi Arabia (*2*). Zoonotic transmission from exposure to MERS-CoV–infected Arabian camels, known as dromedaries, or their raw milk and limited, nonsustained human-to-human transmission have been reported, including large outbreaks in healthcare facilities (*3*–*5*). The recovery of infectious MERS-CoV in virus cultures of specimens from bed sheets, bedrails, intravenous fluid hangers, and radiograph equipment indicates the potential for fomite transmission of the virus in hospitals providing care for MERS-CoV patients (*6*). However, sustained human-to-human transmission has not been documented, and some case-patients have no identified source of exposure to MERS-CoV. As of April 2016, a total of 26 countries had reported locally acquired or exported cases from the Arabian Peninsula, including 2 cases in the United States identified during May 2014 in healthcare personnel who became ill after working in Saudi Arabia (*7*,*8*). A traveler who visited Saudi Arabia, Qatar, the United Arab Emirates, and Bahrain and then returned to South Korea infected with MERS-CoV in mid-2015 triggered 184 MERS-CoV cases, resulting in 38 deaths in multiple health facilities and 1 additional case in a person who traveled to China (*9*,*10*).

Human infections with MERS-CoV are expected to continue to occur on the Arabian Peninsula because of the prevalence of MERS-CoV in dromedaries and the cultural importance of these camels (i.e., for food, milk, and racing purposes) in the region. During the 2003 outbreak of severe acute respiratory syndrome (SARS) in China, civet cats, the suspected reservoir of SARS coronavirus (SARS-CoV), were culled aggressively; no outbreaks were identified after 2004. In contrast, culling of camels is culturally impractical in the Middle East, and MERS-CoV zoonotic infections of humans have continued since 2012.

 The potential for emergence of MERS-CoV mutations that could facilitate sustained community transmission and global dissemination cannot be predicted. No vaccines against or specific treatments for human infection with SARS-CoV, MERS-CoV, or other coronaviruses have been approved. Since 2013, efforts have focused on furthering development of animal models, vaccines, and therapies against MERS-CoV (*11*,*12*). In this report, we update the current state of development for MERS-CoV medical countermeasures, including regulatory challenges in the United States, and draw attention to areas in immediate need of increased infrastructure support for development of these countermeasures.

## Strategies for Potential Use of MERS-CoV Medical Countermeasures

MERS-CoV infection could theoretically be prevented by vaccination, pre- or postexposure antiviral chemoprophylaxis, or passive immunoprophylaxis of persons in affected countries at increased risk for MERS-CoV exposure (e.g., healthcare personnel, persons who work with camels) or persons at higher risk for more severe disease, including persons >65 years of age and those with chronic medical conditions. Therapeutic drugs with specific activity against MERS-CoV (e.g., antiviral drugs, immunotherapeutic treatments) or that target the host immune response could be used for treatment of human illness caused by MERS-CoV infection or for pre- or postexposure prophylaxis. Before human clinical trials of potential MERS-CoV medical countermeasures are started, proof-of-concept data must be obtained from in vivo studies of experimentally infected animals. Such data may indicate a product’s potential efficacy and provide a mechanism for selection of available medical countermeasure candidates. In addition, MERS-CoV vaccines could be developed for animals and used for vaccination of dromedaries on the Arabian Peninsula and in source countries for camel imports to the Horn of Africa to reduce MERS-CoV transmission among camels and possibly from camels to humans.

## Animal Models and Virus Strains

Preclinical development of MERS-CoV medical countermeasures has been hindered by several factors, including limited data on the natural history of MERS-CoV infection in humans; the lack of a small animal model that is naturally susceptible to MERS-CoV; and the inability to consistently replicate severe human disease in MERS-CoV–infected nonhuman primates (NHPs). Another factor is limited access to clinical samples and recent virus isolates; for example, a MERS-CoV strain isolated from a patient in 2012, rather than a more recently isolated strain, is currently used by most investigators worldwide.

Small animal and NHP models are useful for testing potential medical countermeasures for efficacy (Table 1). Studies in mice, both dipeptidyl peptidase-4 (dipeptidyl peptidase-4 or cluster differentiation 26) transduced and transgenic, and in rabbits, hamsters, and ferrets have been reviewed elsewhere (*16*,*20*,*21*). These small animal models have been used for screening potential MERS-CoV medical countermeasures (*13*,*14*,*22*).

**Table 1:** Animal models under development for MERS-CoV, United States*

Source	Species	Genetic modification	Pathology
Perlman Laboratory, University of Iowa, Iowa City, IA	Mouse	Expressing human DPP4 from adenovirus 5 vector	Transient and localized expression of human DPP4, mild infection (*13*)
University of Texas Medical Branch, Galveston, TX	Mouse	Knock-in of human DPP4, constitutive promoter	Expression of human DPP4 throughout the animal, including brain, resulting in relentless weight loss and death within days post infection (*14*)
Regeneron Pharmaceuticals, Inc., Tarrytown, NY	Mouse	Knock-in of human DPP4, natural promoter	Stable expression of human DPP4 under a natural promotor (e.g., limited to the lung, absent in the brain), with viral replication and lung pathology (*15*)
NIAID Rocky Mountain Laboratories, Hamilton, MT; NIH/NIAID/Laboratory of Infectious Diseases, Bethesda, MD	New Zealand white rabbit	Wild-type	MERS-CoV spike protein binds wild-type rabbit DPP4 molecule that allows for attachment and infection by MERS-CoV; intranasal infection leads to mild pulmonary disease and increased viral titers (*16*)
NIAID Rocky Mountain Laboratories	Rhesus macaque	Wild-type	Acute localized to widespread pneumonia with transient clinical disease, similar to mild/moderate human MERS-CoV cases; multifocal, mild to marked interstitial pneumonia, with virus replication occurring mainly in alveolar pneumocytes was observed without evidence of systemic infection (*17*)
NIAID Rocky Mountain Laboratories	Marmoset	Wild-type	MERS-CoV spike protein binds wild-type marmoset DPP4’ multiple routes of infection used; similar to more severe human MERS-CoV cases; lethality observed (*18*)
NIAID Rocky Mountain Laboratories	Dromedaries	Wild-type	Infection studies in a small number of dromedaries underway in a large animal BSL-3 facility in the United States (*19*)

*MERS-CoV, Middle East respiratory syndrome coronavirus; DPP4, dipeptidyl peptidase-4; NIAID, National Institute of Allergy and Infectious Diseases; NIH, National Institutes of Health; BSL-3, Biosafety Level 3.

The major NHP models under development include rhesus macaques and common marmosets (*17*,*18*,*23*). Overall, common marmosets appear to be better suited than rhesus macaques for therapeutic studies designed to target severe disease because marmosets show slightly slower onset of illness and longer duration and severity of disease and their small size requires lower doses of therapeutic drugs. However, the marmoset model has not been standardized and is not consistent between laboratories (*18*,*24*,*25*). Furthermore, the size of marmosets substantially limits sequential blood sampling for virologic or pharmacokinetic testing. Challenges to the development of NHP models include determination and standardization of the optimal MERS-CoV challenge dose and of the volume and route of exposure, as well as the limited availability of NHPs, especially marmosets.

Large animal models in development include camels and camelids such as alpacas (*19*,*26*,*27*). These models may be vital in understanding the virology and immunology of MERS-CoV infection in dromedaries, a natural host. In addition, serologic evidence of MERS-CoV infection in alpacas has been reported in Qatar (*28*). Major gaps for all animal models include a lack of consensus and availability of the optimal animal model to replicate severe human illness from MERS-CoV infection; limited availability of currently or recently circulating MERS-CoV strains; the lack of understanding of clinically relevant symptoms that can be incorporated into clinical scores or used as a signal to begin treatment in animal models; and competition for funding, laboratory space, availability of animals, and expertise with other emerging or reemerging infectious diseases, such as Ebola virus disease and Zika virus disease.

## Diagnostic Devices

Critical issues for facilitating appropriate clinical management of MERS-CoV cases and for implementing infection prevention and control measures in healthcare facilities is the prompt diagnosis of MERS-CoV infection and the monitoring of prolonged viral shedding in severely ill patients and their healthcare and family contacts. Outside of the United States, several commercial and in-house academic laboratory reverse transcription PCR (RT-PCR) molecular assays are available for research, diagnostic, and viral load monitoring purposes. These assays can measure MERS-CoV RNA in samples from symptomatic patients and their asymptomatic contacts. Contributing factors to recent large clusters of MERS-CoV infection in hospitals in Saudi Arabia and South Korea may be linked to inadequate infection-control procedures and prolonged shedding of MERS-CoV. MERS-CoV RNA has been detected for 24–31 days after onset of fever in hospitalized patients (*29*,*30*).

The Secretary of the US Department of Health and Human Services declared a potential public health emergency on May 29, 2013, regarding MERS-CoV infection that could have a high potential to affect national security or the health and security of US citizens living abroad. The US Food and Drug Administration (FDA) subsequently issued an emergency use authorization to the Centers for Diseases Control and Prevention (CDC) for an in vitro molecular diagnostic test to diagnose MERS-CoV infection in multiple types of clinical specimens from symptomatic patients. The use of this test was later expanded to include the ability to test asymptomatic contacts of a person infected with MERS-CoV who traveled from Saudi Arabia to the United States. The CDC made this test available to multiple US public health laboratories, the US Department of Defense, and WHO laboratories worldwide. Although the test has been distributed extensively, it is limited in terms of the CDC’s ability to scale up the supply of reagents to support a surge in MERS-CoV cases in the United States and in other countries where the test has been made available. Therefore, an emergency use authorization was issued on July 17, 2015, for the commercially developed RealStar MERS-CoV RT-PCR Kit U.S. (Altona Diagnostics GmbH, Hamburg, Germany) for use in the in vitro qualitative detection of MERS-CoV RNA in tracheal aspirate or tracheal secretion samples (*31*). Although this commercial assay is a first step in bridging the diagnostic test availability gap in case of a surge scenario, the current coverage, at least in the United States, is insufficient until alternative, FDA-cleared commercial tests are available (Table 2).

**Table 2:** Diagnostics candidates for MERS-CoV*

Source	Method	Status
TIB MolBiol, Berlin, Germany	upE and ORF1a RT-PCR assays	Research use only, not for in vitro diagnostic use; company intent to pursue in vitro diagnostic use unknown (*32*)
Fast-track Diagnostics, Sliema, Malta	hCoV-EMc	In vitro diagnostic for use in the European Community (*33*)
Altona Diagnostics, Hamburg, Germany	RT-PCR Kit	In vitro diagnostic for use in the European Community, FDA EUA (*31*,*33*)
Primerdesign, Chandler’s Ford, UK	Novel Coronavirus hCoV-MERS RT-PCR Kit	Research use only, not for in vitro diagnostic use; company intent to pursue in vitro diagnostic use unknown (*33*)
US Centers for Disease Control and Prevention, Atlanta	Real-time RT-PCR Assay	Available in all US PHL/LRN laboratories and many international governmental laboratories, FDA EUA (*31*)

*MERS, Middle East respiratory syndrome; CoV, coronavirus; upE, upstream of E gene; ORF1a, open reading frame 1a polyprotein; RT-PCR, reverse transcription PCR; hCoV-EMc, Human Coronavirus–Erasmus Medical Center/2012; FDA, US Food and Drug Administration; EUA, emergency use authorization; CDC, US Centers for Disease Control and Prevention; PHL/LRN, Public Health Laboratory/Laboratory Response Network.

A worldwide gap exists in the lack of readily available, simple, rapid, and accurate diagnostic tests for use in outpatient and inpatient clinical settings where the ability of the facility to use currently available, higher complexity molecular tests is limited. The lack of commercial development of MERS-CoV assays may be partially related to the limited availability of clinical specimens and MERS-CoV isolates from infected patients. Availability of serum specimens from RT-PCR–confirmed MERS-CoV patients who survived can help facilitate development of serologic tests. If paired acute and convalescent serum samples are available, serologic tests can be used to confirm MERS-CoV infection when viral shedding is not detectable, and for surveillance purposes such as measuring population exposures and immunity to MERS-CoV infection.

## Therapeutic Drugs

No investigational therapeutic drugs have been evaluated for treatment of MERS-CoV patients in prospective randomized controlled clinical trials. Potential therapeutic drugs for MERS-CoV patients include available approved drugs with nonspecific properties, such as immunomodulators, small-molecule drugs with broad antiviral activity, repurposed FDA-approved small-molecule drugs that show activity against MERS-CoV in vitro (Table 3) (*34*,*35*), and newly developed monoclonal or polyclonal antibody therapies with specific activity against MERS-CoV (Table 4) (*54*).

**Table 3:** MERS-CoV small molecule and biologics treatment candidates*

Source	Drug	Target	Anti–MERS-CoV activity	Status
NIAID Rocky Mountain Laboratories, Hamilton, MT, USA	Ribavirin + IFN	Polymerase + Immunomodulator	Active in cell culture and NHP	Approved for hepatitis C virus, compassionate use for MERS-CoV (*36*–*38*)
University of Hong Kong, Hong Kong	Interferon B1b	Immunomodulator	Active in cell culture	Preclinical development (*24*)
Hemispherix Biopharma, Philadelphia, PA, USA	Alferon N	Immunomodulator	Active in cell culture	Approved for human papillomavirus, orphan drug designation granted by the European Medicines Agency (*39*)
Romark Laboratories, Tampa, FL, USA	Nitazoxanide	Host functions, glycosylation	Active in cell culture	Approved for cryptosporidia and giardia, in clinical trials for influenza virus (*40*)
AbbVie, North Chicago, IL, USA	Lopinavir	Protease	Active in cell culture, NHP models	Approved for HIV (*24*)
BioCryst Pharmaceuticals, Durham, NC, USA	BCX4430	Polymerase	Active in cell culture and (Ad5) DPP4 mouse	Clinical trial for Ebola virus (*41*)
Sarafianos Laboratory, Columbia, MO, USA†	SSYA10–001	Helicase	Active in cell culture	Broadly active against coronaviruses (*42*)
Planet Biotechnology, Hayward, CA, USA	Immunoadhesin (DPP4-Fc)	Spike/binding	Active in cell culture	Preclinical development (*43*)
New York Blood Center, New York, NY, USA	HR2P-M2	Spike/fusion	Active in mouse models	Preclinical development (*44*)
Loyola University, Chicago, Stritch School of Medicine, Maywood, IL, USA	Protease inhibitors	MERS-CoV PLpro, MERS-CoV 3CLpro5	Active in cell culture	Preclinical development (*45*)
University of Maryland, College Park, MD; Rega Institute, Katholieke Universiteit Leuven, Belgium; NCATS; NIAID; University of Leiden, South Holland, The Netherlands	FDA-approved drug screens	Multiple host targets	Active in cell culture; chloroquine and chlorpromazine are promising	Multiple screening efforts (*34*,*35*)

*MERS-CoV, Middle East respiratory syndrome coronavirus; NIAID, National Institute of Allergy and Infectious Diseases, National Institutes of Health; INF, interferon; NHP, nonhuman primate; DPP4, dipeptidyl peptidase-4; spike, MERS-CoV spike protein; PLpro, papain-like protease; 3CLpro, 3C-like protease; NCATS, National Center for Advancing Translational Sciences, NIAID; FDA, US Food and Drug Administration. †Christopher Bond Life Sciences Center, University of Missouri Department of Molecular Microbiology and Immunology, University of Missouri School of Medicine.

**Table 4:** MERS-CoV immunotherapeutic treatment candidates*

Source	Drug	Target	Anti–MERS-CoV activity	Status
Multiple	IVIG	Spike, immune system	Unknown	Intravenous immunoglobulin is available and has been used for the treatment of >1 MERS-CoV patients with unknown clinical benefit (*40*)
King Abdullah International Medical Research Center, Riyadh, Saudi Arabia	Convalescent serum	Spike, immune system	Ad5-DPP4 mouse efficacy	A pilot clinical trial of convalescent plasma treatment of MERS-CoV patients is ongoing but not recruiting in Saudi Arabia (*46*)
Sanford Applied Biosciences, Sioux Falls, SD, USA	Transgenic bovine polyclonal	Spike	Ad5-DPP4 mouse and NHP studies	Preclinical development (*47*)
National Cancer Institute, NIH, Bethesda, MD, USA	M336, M337, M338	Spike	MERS-CoV neutralization	Preclinical development (*48*)
Tsinghua University, Beijing, China	MERS-4, MERS-27	Spike	MERS-CoV neutralization	Preclinical development (*49*)
Dana Farber Institute, Boston, MA, USA	3B11, 1F8, 3A1, 80R	Spike	MERS-CoV neutralization	Preclinical development (*50*)
New York Blood Center, New York, NY, USA	Mersmab1	Spike	MERS-CoV neutralization	Preclinical development (*51*)
Regeneron Pharmaceuticals, Tarrytown, NY, USA	REGN3051, REGN3048	Spike	MERS-CoV neutralization and humanized DPP4 mouse studies	Preclinical development (*22*)
Juntendo University, Tokyo, Japan	2F9 and YS110	CD26	VLP neutralization	Preclinical development (*52*)
Humabs Biomed SA, Bellinzona, Switzerland	LCA60	Spike	Ad5-DPP4 mouse	Preclinical development (*53*)

*MERS-CoV, Middle East respiratory syndrome coronavirus; spike, MERS-CoV spike protein; MG, immunoglobulin; Ad5-DPP4, adenovirus 4 virus expressed dipeptidyl peptidase-4; NHP, nonhuman primate; DPP4, dipeptidyl peptidase-4; CD26, dipeptidyl peptidase-4; VLP, virus-like particle.

One promising approach has been to investigate libraries of drugs approved by the FDA and the European Medicines Agency. Considering development times and manufacturing requirements for new products, repurposing of existing drugs might potentially facilitate a rapid response to outbreaks of emerging viruses (see Regulatory section for a discussion on repurposing). Other early-stage work on MERS-CoV therapeutics includes studies focusing on the essential viral replication steps of fusion, proteolysis, and RNA polymerization (Table 3) (*54*).

Immunotherapeutics under evaluation consist of convalescent plasma and monoclonal and polyclonal antibodies. Most of the monoclonal antibodies in development have specific neutralizing activity against the MERS-CoV spike protein (*55*,*56*). Platforms are being developed to rapidly discover monoclonal antibodies, either from fully human convalescent blood or from transgenic animals, which can be manufactured on a large scale and are likely to have a good safety profile. The most advanced immunotherapeutic for MERS-CoV uses a transchromosomal bovine production system to produce fully human polyclonal MERS-CoV antibodies; a phase I study of this produce was recently implemented (*57*; https://clinicaltrials.gov/ct2/show/NCT02788188). Preliminary results from immunoprophylaxis or treatment studies have shown efficacy of fully human monoclonal or polyclonal antibodies in MERS-CoV-infected mice and NHPs (Table 4). Although fully human monoclonal antibodies typically have a good safety profile and a defined set of preclinical toxicology studies, challenges to development of immunotherapeutics include ensuring the absence of antibody-dependent enhancement of disease and reducing the risk for generation of escape mutant viruses that would be resistant to treatment.

## Vaccines

### Human Vaccination

Development of MERS-CoV candidate vaccines was initiated by the National Institute for Allergy and Infectious Diseases at the National Institutes of Health, academic investigators, and several companies (Table 5). Most candidate vaccines are still being evaluated in animal models. They have generally targeted the spike protein of MERS-CoV and are recombinant virus, subunit, DNA, or virus-like vector vaccines (*60*,*63*–*67*). One live-attenuated MERS-CoV candidate vaccine is in early development (*66*). Preliminary studies for several other MERS-CoV vaccine candidates have been initiated, and early results demonstrate immunogenicity; 2 have progressed to NHP challenge, and a phase 1 clinical study in adults of 3 different doses of a DNA plasmid vaccine that expresses the MERS-CoV spike protein was started in January 2016 (*61*). Ongoing assessment of antigenic evolution of circulating MERS-CoV strains is essential for informing vaccine development (*68*).

**Table 5:** Human vaccine candidates for MERS-CoV targeting spike protein*

Source	Vaccine	Status
Novavax, Gaithersburg, MD, USA	Spike protein trimer in 40 nm particle; likely adjuvanted	Mouse-immunogenicity shown (*58*)
NIAID/Vaccine Research Center, Bethesda, MD, USA	Two candidate vaccine approaches: DNA spike prime-S1 protein boost and S1 prime-S1 boost	Mouse and NHP immunogenicity shown; NHP^2^ (macaque-radiological efficacy shown) (*59*)
GeneOne Life Science, Seoul, South Korea; Inovio Pharmaceuticals, Plymouth Meeting, PA, USA	DNA expressing spike; electroporation device	Mouse, NHP, and camel immunogenicity shown; NHP^2^ (viremia, lung pathology) (*60*); phase I study started (*61*)
Greffex, Aurora, CO, USA	Fully deleted adenovirus packaging vector	Mouse immunogenicity (*62*)
Erasmus University Rotterdam, The Netherlands; University of Marburg, Marburg; Ludwig-Maximilians University, Munich, Germany	MVA vectored spike protein	Mouse immunogenicity and protection shown; clinical trials in planning stage (*63*,*64*)
New York Blood Center, New York, NY, USA; Shanghai Medical College, Shanghai, China	Spike receptor-binding domain subunit vaccine	Recombinant protein containing the 377–588 aa fragment of the S1 subunit (*65*)

*MERS-CoV, Middle East respiratory syndrome coronavirus; spike, MERS-CoV spike protein; NHP, non -human primate; MVA, modified vaccinia Ankara; S1, portion of spike protein with the receptor binding domain.

A concern that must be addressed in the development of MERS-CoV vaccines is the potential for causing antibody-dependent enhancement of disease upon virus challenge, such as what was observed with a SARS-CoV candidate vaccine upon SARS-CoV challenge (*69*). The lack of a precedent of coronavirus vaccines for humans poses another challenge for the evaluation of MERS-CoV vaccines for humans, although vaccines against other animal coronaviruses are safe and in use in animals.

### Camel Vaccination

Considering the cultural importance of dromedaries on the Arabian Peninsula for meat, milk, and racing, prevention of camel-to-camel MERS-CoV transmission and reduction of spread from dromedaries to humans by camel vaccination is being investigated by government, academic, and commercial investigators (Table 6). Young camels appear to be at high risk for MERS-CoV infection and could be a priority group for vaccination (*73*,*74*); the loss of maternal MERS-CoV antibodies ≈5–6 months after birth suggests a short time window for vaccination (*85*). A major challenge to this approach is that dromedaries can be reinfected with MERS-CoV; a study by Farag et al. found no correlation between MERS-CoV RNA levels and neutralizing antibodies in camels (*75*), suggesting that antibodies may not be protective against infection. Because older camels can be reinfected, a camel vaccination strategy may require multiple dosing and booster vaccination to increase effectiveness over time. Experimental MERS-CoV infection studies and vaccine studies of a small number of dromedaries have been conducted in large animal Biosafety Level 3 facilities in the United States and overseas (*19*). In addition, 3 doses of a DNA vaccine containing the MERS-CoV spike protein induced humoral immunity in dromedaries (*60*). In a recent study, a modified vaccinia virus Ankara vaccine that expresses the MERS-CoV spike protein was administered intranasally and intramuscularly to dromedaries; when challenged intranasally with MERS-CoV, vaccinated dromedaries had fewer signs of respiratory infection and lower MERS-CoV titers in the upper respiratory tract compared with unvaccinated dromedaries (*76*). Alpacas (New World camelids) are being investigated as a suitable proxy for camels because of the lack of available dromedaries in the United States, the high cost of acquiring dromedaries, and the relatively smaller size of alpacas (*26*,*27*).

**Table 6:** Camel vaccine candidates for MERS-CoV targeting spike protein*

Source	Vaccine	Status
USG/Academic Institution Consortium	Recombinant and inactivated whole virus	Camel vaccination
NIAID Rocky Mountain Laboratories, Hamilton, MT, USA/Colorado State University, Fort Collins, CO, USA	Spike protein subunit vaccine/Advax adjuvant (baculovirus expressed)	Camel and alpaca vaccination studies (*70*,*71*)
Erasmus University Rotterdam, The Netherlands; University of Marburg, Marburg; Ludwig-Maximilians University, Munich, Germany	MVA-vectored spike protein	Camel vaccination challenge studies (*71*,*72*)
Novavax AB, Uppsala, Sweden	Spike nanoparticles with adjuvant likely	In preclinical development
University of Pittsburgh, Pittsburgh, PA	Adenovirus vectored spike protein	In preclinical development (*72*)

*MERS-CoV, Middle East respiratory syndrome coronavirus; spike, MERS-CoV spike protein; USG, US government; NIAID, National Institute of Allergy and Infectious Diseases, National Institutes of Health; MVA, modified vaccinia Ankara.

## Regulatory Considerations for Medical Countermeasures in the United States

Regulatory considerations for MERS-CoV medical countermeasures in the United States are focused on a pathway to human clinical trials for drugs and vaccines through submission of investigational new drug applications. Investigational new drug submissions must adhere to requirements set forth in the Code of Federal Regulations, Title 21, Part 312 (21 CFR 312; http://www.ecfr.gov/cgi-bin/text-idx?tpl=/ecfrbrowse/Title21/21cfr312_main_02.tpl). Several guidance documents exist on the FDA website related to virology, microbiology, pharmacology and toxicology, and clinical and medical considerations (*77*). The most appropriate approval pathway is likely to be product-specific and will require consideration of existing product data, proposed intended use and population for use, and validated endpoints for efficacy predictive of clinical benefit, if any. Likewise, data needed for consideration of an emergency use authorization, including dose finding and dose ranging, duration, and safety, can be obtained through sources such as investigational new drug clinical trials.

Repurposing of drugs approved by the FDA for other illnesses for a MERS-CoV indication can potentially be expedited or accelerated if 1) the mechanism of action for antiviral activity is defined, 2) there is no change to the approved final drug form and route of administration, 3) dosing does not exceed the currently approved dose and duration for the currently indicated population and adequate pharmacokinetics data support this dosing, and 4) the risk–benefit profile is acceptable for the intended population and indication. For example, the risk–benefit profile for an approved drug with an oncology indication may be unacceptable if the drug is repurposed for administration to a healthy population for MERS-CoV postexposure prophylaxis. However, data requirements to initiate human trials will depend on the characteristics of the drug product and its intended use against MERS-CoV. As such, sponsors should consider prioritizing drug development on the basis of the totality of scientific evidence and merit of the drug alone, not on whether the drug has been previously approved.

In the absence of a standardized and accepted animal model that simulates human disease from MERS-CoV infection, it is unclear how the FDA may be able to expedite licensure or approval when data are lacking. The best approach may be collection of preclinical safety data and implementation of adaptive human clinical trials. This approach was taken for medical countermeasures in response to the 2013–2016 Ebola virus disease outbreak.

For diagnostic devices, the current emergency use authorization pathway serves as a fast approach to make products available for emergency public health purposes. After an emergency has been terminated, Premarket Notifications for these products should be submitted to FDA for a more thorough evaluation as 510(k)s (http://www.fda.gov/MedicalDevices/ProductsandMedicalProcedures/DeviceApprovalsandClearances/510kClearances/default.htm).

## Clinical Experience and Medical Countermeasure Trials

The overarching goal for clinical research of MERS-CoV patients is to optimize clinical management and to identify effective therapies to improve survival. Although clinical data on some MERS-CoV patients have been published in case series (*58*,*78*,*79*), there is a need for much more epidemiologic, clinical, virologic, and immunologic data to improve the limited understanding of the pathogenesis of MERS-CoV infection in humans. Gaps include information on viral load and duration of viral shedding in blood, urine, respiratory, and other clinical specimens from infected persons; understanding of the innate and adaptive immune response to MERS-CoV infection; pathology data on the distribution of MERS-CoV in respiratory and extrapulmonary tissues in fatal cases; information from autopsies of persons who died of MERS-CoV; and an overall improved understanding of the pathogenesis of MERS-CoV in humans. One study investigated MERS-CoV infection in autopsy tissues of a patient who died from the disease (*80*). Collaborations are especially needed to pool and systematically collect serial clinical specimens from MERS-CoV patients for virologic, immunologic, and biomarker analyses to correlate with clinical illness, and to conduct long-term follow-up of survivors of severe disease (*81*–*83*). Detailed understanding of host factors and cofactors associated with disease severity from asymptomatic infection to fatal illness is needed. Efforts to promote international sharing of clinical specimens and MERS-CoV isolates are needed to foster development of therapeutics and vaccines.

Use of standardized clinical data collection instruments and common biologic sampling protocols for serial prospective data collection will facilitate data pooling from MERS-CoV cases and comparisons across clinical sites and countries. Global collaborations among clinical networks are also needed to implement clinical trials, preferably randomized controlled clinical trials, of MERS-CoV investigational therapeutics, (*81*–*84*). Without an international agreement on protocols and systematic standardization of case reporting and data collection methods, haphazard or anecdotal reporting and analysis of disease course and outcome may continue. WHO and the International Severe Acute Respiratory and Emerging Infection Consortium are collaborating in adapting standardized protocols for controlled clinical trials for MERS-CoV (*82*).

## Timelines for Clinical Trials of MCMs

Prospective controlled clinical trials (ideally randomized clinical trials) of potential MERS-CoV therapies and vaccines in humans are needed urgently; however, there is uncertainty in estimating timelines for the development of potential MERS-CoV medical countermeasures because of the need to further characterize existing and new animal models, the unpredictability of demonstrating a favorable risk–benefit outcome during preclinical testing, and competition for resources with other emerging infectious diseases. In addition, the risk for antibody-dependent enhancement of disease may interrupt the timeline for conducting human clinical trials of MERS CoV vaccines and immunotherapeutics. Researchers of all potential MERS-CoV medical countermeasures should have preclinical toxicology data available before initiating human clinical trials. Although animal efficacy data are not technically required before implementing human clinical trials of potential countermeasures, such data are considered important for identifying the most promising medical countermeasure candidates, justifying risk in human volunteers, and informing the design of future clinical studies. Timeframes for the production of specimen panels and repositories to aid commercial diagnostic development are also contingent on obtaining adequate funding and clinical samples.

## Conclusions

Although preclinical development and research on potential MERS-CoV medical countermeasures has achieved appreciable progress to date, such development is preliminary, and substantive challenges must be overcome before most potential countermeasures are ready for human clinical trials. The only clinical trials of MERS-CoV medical countermeasures are phase I studies of a candidate vaccine and an immunotherapeutic that are ongoing. Prioritization of animal models, standardization of representative virus strains, and establishment of clinical trial capabilities in areas where the virus is endemic among dromedaries are viewed as critical elements of effective MERS-CoV medical countermeasures development. Results of substantial progress in establishing the infrastructure and platforms for preclinical and advanced clinical development of countermeasures can serve as a model to enable more timely response to other emerging infectious diseases of global public health concern in the future.
